# Mapping Women-Only Health Research in Taiwan: A Bibliometric Analysis of Research Trends, Collaboration, and Thematic Clusters (2015–2025)

**DOI:** 10.3390/healthcare14172842

**Published:** 2026-09-03

**Authors:** Nirmin F. Juber, Manal Taimah, Hsiao-Feng Cheng, Yuen-Hsien Tseng

**Affiliations:** 1 Department of Health Promotion and Health Education, National Taiwan Normal University, Taipei 106308, Taiwan; 2 Public Health Research Center, New York University Abu Dhabi, Abu Dhabi 129188, United Arab Emirates; mkt6@nyu.edu; 3 Department of Educational Psychology and Counseling, National Taiwan Normal University, Taipei 106308, Taiwan; cindy6510@ntnu.edu.tw; 4 Graduate Institute of Library & Information Studies, National Taiwan Normal University, Taipei 106308, Taiwan; samtseng@ntnu.edu.tw

**Keywords:** women’s health, bibliometric analysis, Taiwan, public health, Scopus

## Abstract

**Background**: Research on women’s health is essential for addressing sex-specific healthcare needs and informing evidence-based policy. However, women-only health research in Taiwan has not been systematically examined. **Methods**: We conducted a Scopus-based bibliometric analysis of health research exclusively involving female populations in Taiwan from 2015 to 2025. We included English-language, peer-reviewed journal articles involving only female participants and conducted in Taiwan or using Taiwanese datasets. Mixed-sex studies, including those reporting female-specific or sex-stratified findings, were excluded. Publication trends, citations, international collaboration, secondary data sources, and thematic patterns were analyzed using Biblioshiny and VOSviewer. **Results**: A total of 1345 publications across 446 journals were included. Publication output increased from 126 articles in 2015 to a peak of 153 in 2021, followed by a decline, with an annual growth rate of −0.4%. The publications involved 3412 authors and received an average of 14.52 citations per document. International co-authorship accounted for 19.48% of publications, with the United States as the leading collaborator. The National Health Insurance Research Database was the most frequently reported secondary data source (n = 371, 27.6%). Research was concentrated in cancer, reproductive health, and mental health, whereas cardiovascular health accounted for a comparatively smaller share of the literature. **Conclusions:** Women-only health research in Taiwan remained active throughout the study period, despite a decline in publication output after 2021. The extensive use of national health databases and international collaboration further characterized the research landscape. These findings provide an evidence-informed basis for future research priority-setting, including efforts to broaden research across women’s health conditions and life-course stages while considering research activity alongside population health needs. Integrating bibliometric evidence with population health priorities may support a more balanced and comprehensive women’s health research agenda in Taiwan.

## 1. Introduction

Women’s health extends beyond reproductive function and encompasses biological, psychological, environmental, and social well-being across the life course [1]. Women have long been underrepresented in health studies despite their distinct physiological, psychological, and social characteristics [2]. As a result, study findings from male or mixed-sex populations have frequently been generalized to women, potentially overlooking sex-specific risk factors, disease manifestations, treatment effectiveness, and healthcare needs [3,4]. Despite the growing volume of women-focused research recently, disparities in research attention across health domains remain evident [5]. While progress has been made in areas such as mental health research, women-focused research remains relatively limited in cardiovascular health [5,6], despite cardiovascular diseases (CVDs) accounting for approximately one-third of deaths among women globally [7].

Supported by a universal national health insurance system and comprehensive national health databases, Taiwan provides a strong foundation for population health research [8,9,10]. These nationally representative data resources have facilitated extensive research on women’s health across clinical, epidemiological, and public health disciplines. Women account for nearly half of Taiwan’s population [11] and continue to face distinct health challenges across the life course. These include a high burden of breast and gynecological cancers [12,13], reproductive health issues and infertility [14], mental health disorders [15], menopause-related conditions [16], and the growing impact of chronic diseases among an aging female population [17]. In addition, rapid social changes, increasing female workforce participation, changing family structures, and demographic transitions [18] have created new health and healthcare needs for women in Taiwan. Although these issues have been investigated across individual health conditions and population groups, the overall distribution and structure of research conducted exclusively among women have not been systematically characterized.

As the volume of the scientific literature grows, it becomes increasingly difficult to obtain a comprehensive view of research productivity, collaboration patterns, influential publications, and emerging themes in a field. Bibliometric analysis provides a systematic and quantitative way to map the structure and evolution of scientific knowledge [19]. Bibliometric methods can reveal research strengths, emerging priorities, and knowledge gaps [20]. Importantly, mapping women-only research provides a population-specific perspective that complements existing disease-specific studies and research based on mixed-sex populations. While studies of individual conditions can describe specific health problems, they do not necessarily show how research attention is distributed across health domains, life-course stages, or research themes, nor how research capacity and collaboration are structured across the field. Despite the increasing volume of health-related research in Taiwan, no previous bibliometric study has systematically mapped research conducted exclusively among women in Taiwan. Consequently, it remains unclear which research areas have received greater or less research attention, which datasets and research resources have been most frequently used, how researchers and institutions collaborate, and how research themes have changed over time.

A systematic mapping of this research landscape can provide an evidence base for identifying areas of research concentration, informing future research directions and facilitating more coordinated research efforts. It may also help researchers, research institutions, and funding agencies recognize areas warranting further investigation and opportunities for interdisciplinary and international collaboration. However, publication volume alone does not establish the adequacy of research relative to disease burden or healthcare needs; rather, bibliometric findings provide a description of research activity that can complement epidemiological and policy evidence.

The present study aimed to provide a Scopus-based bibliometric analysis of women-only health research in Taiwan from 2015 to 2025. Specifically, we examined: (1) publication and citation characteristics as well as international collaboration patterns; (2) the use of data sources and secondary databases; and (3) major research themes, emerging topics, and their temporal patterns in women-only health research in Taiwan. By addressing these dimensions, the study seeks to provide a systematic overview of the research landscape and identify areas of concentrated and comparatively limited research activity that may inform future research and policy discussions on women’s health in Taiwan.

## 2. Methods

This bibliometric study examined publication trends, research characteristics, international collaboration patterns, and thematic structure of women-only health research conducted in Taiwan and published in Scopus-indexed journals between 2015 and 2025. In this study, women-only health research was defined as original research involving exclusively female participants and addressing health conditions, determinants, or outcomes relevant to women across the life course, including reproductive and maternal health, gynecological conditions, cancer, chronic diseases, mental health, psychosocial well-being, and other health conditions affecting women [1].

The study used a structured, concept-based search strategy to maximize the retrieval of potentially relevant publications. The search strategy combined terms representing women or female populations with keywords covering major domains of women’s health across the life course and terms identifying Taiwan. The selection of keywords was informed by previous women’s health literature, Medical Subject Headings, and internationally recognized women’s health frameworks. The search covered reproductive and maternal health, gynecological disorders, female-specific cancers, mental health, endocrine disorders, cardiovascular diseases, neurological disorders, autoimmune diseases, and other major non-communicable diseases affecting women. This concept-based approach was adopted to improve retrieval sensitivity while maintaining relevance to the study objective of mapping women-only health research. The broader search yielded a thematic distribution that was highly consistent with that of the primary search, providing additional support for the robustness of the search strategy.

To maintain consistency and methodological robustness in bibliometric mapping, this study focuses exclusively on English-language journal publications. The analysis was limited to English-language journal publications, as these are more consistently indexed in Scopus [21]. These publications generally have greater international visibility and more standardized bibliographic metadata [21,22], including abstracts and authors’ keywords, which are crucial for enhancing the reliability of co-occurrence and network analyses [23]. In bibliometric analysis, publication output is commonly used as a proxy for academic productivity [19], with bibliographic records extracted from a database and analyzed using quantitative and qualitative approaches. Several common databases are suitable for bibliometric analysis, including Scopus and Web of Science [24]. In this study, we used Scopus, an Elsevier database, to retrieve publications on women’s health in Taiwan. This database was selected for several reasons, including extensive indexing and bibliometric analysis tools [25], and broad coverage of the peer-reviewed literature [24]. The study period was set from 2015 to 2025 to provide a decade’s perspective on research trends, as this period aligns with the implementation of the United Nations Sustainable Development Goals (SDGs), particularly SDG 3 (Good Health and Well-being) and SDG 5 (Gender Equality).

### 2.1. Definition and Operationalization of Women-Only Health Research

For this study, women-only health research was operationalized as original peer-reviewed research that exclusively included female participants and investigated health conditions, determinants, or outcomes relevant to women across the life course. Women-only status was determined based on the study population reported in each publication and was not inferred solely from the presence of female-related terminology in the bibliographic record. Eligible research included studies of reproductive and maternal health, gynecological conditions, cancer, non-communicable diseases, mental health, psychosocial well-being, and other health issues affecting women. Studies involving both male and female participants were excluded, even when female-specific or sex-stratified analyses were reported. This criterion was applied to maintain conceptual consistency and ensure that the analysis represented research conducted exclusively among women rather than research in which women constituted one subgroup of a mixed-sex population. Consequently, the resulting research landscape represents the body of health research exclusively conducted among female populations and should not be interpreted as representing the complete body of research that generates evidence relevant to women’s health. In particular, studies using mixed-sex populations that reported sex-specific findings were outside the scope of this analysis.

### 2.2. Search Strategy

A structured search strategy was developed to identify potentially relevant publications in Scopus. Rather than applying restrictive participant-related filters at the database-search stage, the search was intentionally broad because information on participant sex, age, and population composition is not consistently available in bibliographic indexing fields. The search combined three concept groups: (1) terms representing women or female populations; (2) terms representing major women’s health conditions and domains; and (3) terms identifying Taiwan. The final search string was the following:

(TITLE-ABS-KEY(abortion OR “assisted reproductive*” OR IVF OR “breast cancer” OR breastfeeding OR “breast-feeding” OR “cancer screening” OR “cardiovascular disease*” OR CVD* OR “cervical cancer” OR cesarean OR caesarean OR “colorectal cancer” OR “endocrine reproductive disorders” OR “endometrial cancer” OR endometri* OR estrogen OR “female health” OR “female reproduction” OR fertility OR fibroids OR “geriatric women” OR gestational OR gestation* OR gynaecolog* OR gynecolog* OR “heart disease” OR “heart diseases” OR “hormone replacement therapy” OR HRT OR “human papillomavirus vaccine” OR HPV OR “hyperglycemic women” OR hysterectomy OR “immigrant women” OR “immigrant health” OR infertility OR lactation OR leiomyomas OR mammogra* OR “maternal suicide” OR matern* OR menarche OR menopause OR “menstrual distress” OR menstrual OR menstrua* OR miscarriage OR obstetric* OR ovar* OR “ovarian carcinoma” OR “ovarium cancer” OR “papanicolaou test” OR “pap smear” OR “pelvic inflammatory disease” OR PID OR perimenopaus* OR perinatal OR “perinatal depress*” OR postmenopausal OR postmenopaus* OR post-menopaus* OR postnatal OR “postpartum depression” OR postpartum OR “polycystic ovari*” OR PCOS OR “pre-pregnancy” OR preterm OR pre-term OR preconceptional OR preeclampsia OR premenopaus* OR prenatal OR pregnan* OR “Taiwan maternal and infant” OR “reproductive disorders” OR “reproductive-aged women” OR stillbirth OR “systemic lupus erythematosus” OR SLE OR uter* OR “uterine fibroids” OR “women health” OR thyroid OR “thyroid disease*” OR osteoporosis OR migraine OR headache OR “chronic disease*” OR “non-communicable disease*” OR “noncommunicable disease*” OR NCD*)) AND (TITLE-ABS-KEY(women OR woman OR female* OR maternal OR pregnant)) AND (TITLE-ABS-KEY(Taiwan*)) AND PUBYEAR > 2014 AND PUBYEAR < 2026 AND (LIMIT-TO (DOCTYPE, “ar”)) AND (LIMIT-TO (LANGUAGE, “English”)).

The search was conducted on 30 June 2026. The search strategy was designed to maximize retrieval sensitivity and therefore did not use participant sex, age group, or population composition as restrictive database filters. These characteristics were verified during subsequent manual screening rather than inferred from Scopus indexing terms. Importantly, the presence of female-related or disease-specific terms in the title, abstract, or keywords was not considered sufficient to establish a women-only study. An additional search using broader generic women’s health terms, “Women’s Health” OR “Female Health”, was conducted as a sensitivity analysis. The thematic distribution identified using the broader search was highly consistent with that of the primary search, providing additional support for the robustness of the concept-based search strategy.

#### 2.2.1. Inclusion Criteria

Studies were eligible if they met all of the following criteria:

- Original, peer-reviewed journal articles published between 2015 and 2025.
- Studies involving exclusively female participants.
- Research related to women’s health conducted in Taiwan through primary data collection, including studies involving Taiwanese and non-Taiwanese participants in Taiwan, or through secondary data analysis using Taiwanese datasets.
- Research addressing health conditions, determinants, behaviors, or outcomes relevant to women’s health.
- Quantitative or qualitative original research published in English-language journals.

#### 2.2.2. Exclusion Criteria

Records were excluded if they met any of the following criteria:

- Studies involving mixed-sex or male-only populations, non-human subjects, infants, children, or adolescents.
- Reviews, systematic reviews, meta-analyses, conference abstracts, guidelines, editorials, letters, case reports, case studies, and other non-original publications.
- Studies that did not involve Taiwan, Taiwanese populations, or Taiwanese datasets.
- Studies outside the scope of medical, health, or public health research.
- Non-English publications and English-language in-press articles.

### 2.3. Study Selection and Data Processing

To maximize retrieval sensitivity, we conducted a broad Scopus search without applying restrictive participant-related exclusion terms in the initial Boolean search. The initial search identified 7873 records. After retrieval, Scopus’s built-in filters were applied sequentially to efficiently reduce records that were outside the scope of the study. These database-level filters were used solely to reduce the retrieval set and were not considered substitutes for the subsequent manual assessment of study eligibility. First, the document-type filter excluded 10 in-press articles, leaving 7863 records. Second, the keyword filter excluded 5955 records containing indexed keywords indicative of populations outside the target population, including “adolescent,” “animal,” “animals,” “child,” “child-preschool,” “infant,” “infant-newborn,” “male,” “newborn,” and “nonhuman,” leaving 1908 records. Third, the field-category filter excluded 220 records from unrelated subject areas (i.e., non-health research). Finally, the country filter excluded 111 records associated with countries other than Taiwan. These sequential Scopus filters reduced the retrieval set from 7873 to 1577 records.

**Figure 1:**
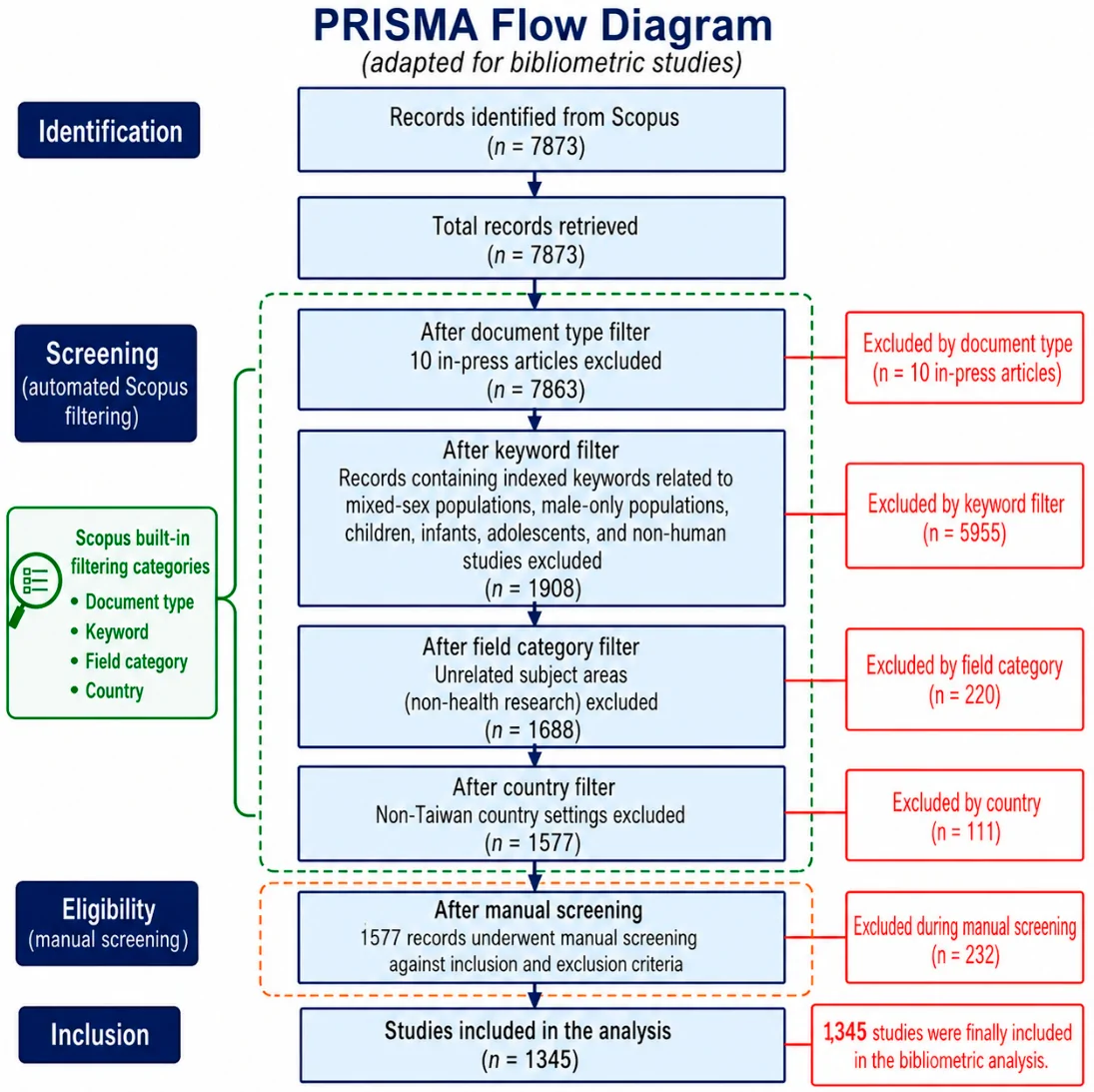
PRISMA flowchart of the study selection process.

The 1577 remaining records were imported into Rayyan for manual screening against the predefined inclusion and exclusion criteria. One researcher performed the primary screening of all 1577 records using Rayyan to facilitate the screening workflow. Titles and abstracts were assessed first, followed by full-text review when necessary. During manual screening, records were assessed in greater detail with respect to participant characteristics, study setting and country, research field, and other predefined eligibility criteria. Records in which Taiwan was mentioned only incidentally, rather than representing the study setting, study population, or data source, were also excluded. A total of 232 records were excluded during manual screening, resulting in 1345 eligible studies for inclusion in the bibliometric analysis (Figure 1).

To assess inter-rater reliability, a second researcher independently screened a randomly selected sample of 173 records from the 1577 records subjected to manual screening using Rayyan. Cohen’s kappa indicated substantial agreement between the two researchers (κ = 0.864, 95% CI: 0.77–0.95; *p* < 0.001). Any disagreements or uncertainties were discussed and resolved by consensus with reference to the predefined eligibility criteria. Rayyan was used to facilitate the screening workflow, including AI-assisted prioritization of records. AI-generated recommendations were used solely to prioritize the screening order and did not determine eligibility or exclusion decisions. All final eligibility decisions were made by the researchers.

### 2.4. Bibliometric and Thematic Analysis

The primary bibliometric analysis was designed to address three analytical dimensions: (1) publication and citation characteristics and international collaboration patterns; (2) the use of data sources and secondary databases; and (3) thematic structure, research topics, and their temporal patterns. A separate post-hoc exploratory analysis was subsequently conducted to assess cardiovascular research representation. Bibliographic data from the 1345 included articles were analyzed using Biblioshiny (version 5.0), within RStudio (version 2026-04-24), and VOSviewer version 1.6.21. Descriptive bibliometric indicators were generated using Biblioshiny, including annual scientific production, publication sources, authorship patterns, citation metrics, institutional productivity, and international collaboration.

#### 2.4.1. Publication and Collaboration Characteristics

Publication trends were assessed using annual scientific production. Citation indicators, including citation counts and average citations per document, were summarized descriptively, together with leading journals, productive authors and institutions, and authorship patterns. We did not calculate the h-index or self-citation rates because the study focused on describing publication output, collaboration, data source utilization, and thematic structure rather than evaluating citation performance or individual research impact. International collaboration was examined at the country level using co-authorship information derived from author affiliations. These analyses were used to characterize the overall structure and productivity of women-only health research in Taiwan.

#### 2.4.2. Secondary Data Source Utilization

Secondary data source utilization was assessed by identifying the named datasets reported in the included publications. Secondary databases were categorized according to the specific national or population-based data source used, and the number and proportion of publications using each dataset were calculated descriptively. Publications using more than one secondary data source were counted for each dataset reported.

#### 2.4.3. Thematic Structure

Authors’ keywords were analyzed to identify the major thematic domains represented in the literature. Biblioshiny was used to generate a thematic map based on keyword co-occurrence. The thematic map was constructed using the following parameters: the top 200 most frequent words, a minimum cluster frequency of 5 per 1000 documents, an alpha parameter of 0.5 to balance relevance and centrality, and community repulsion of 0.5. Communities were identified using the Louvain clustering algorithm. The thematic map was used to identify and characterize major research domains according to their centrality and density. Centrality reflects the degree of a theme’s connections with other themes, whereas density reflects the internal development and coherence of the theme. The size of each cluster represents its relative frequency within the dataset. This analysis provided an overview of the thematic structure of women-only health research in Taiwan.

#### 2.4.4. Research Topics and Temporal Trends

Changes in research topics over time were assessed using Biblioshiny’s trend-topic analysis. A minimum keyword frequency of 5 and a minimum of 3 words per year were applied. The frequency threshold of 5 was selected to retain recurring topics while excluding terms with very limited occurrence, thereby providing a sufficiently broad representation of research topics across the study period. This analysis was used to identify frequently occurring topics and examine their distribution over time, providing evidence of changes in research emphasis.

#### 2.4.5. Keyword Co-Occurrence Network

A keyword co-occurrence network was generated using VOSviewer with the following strategies: 1. counting methods: full accounting, 2. normalization procedures: association strength, 3. clustering parameters: resolution of 1.00 and minimum size of 1, 4. minimum occurrence thresholds: 30, and keyword harmonization or cleaning procedures: manual filtering and selection. The minimum occurrence threshold of 30 was applied to focus the network visualization on the most frequently occurring keywords and reduce network complexity, thereby improving the interpretability of the resulting co-occurrence structure. This higher threshold, compared with the minimum keyword frequency of 5 used in Biblioshiny’s trend-topic analysis, reflected the different purposes of the two analyses: Biblioshiny was used to provide a broader assessment of thematic patterns and topic trends, whereas VOSviewer was used to visualize the dominant structure and relationships among frequently co-occurring keywords. Therefore, the sets of keywords represented in the two analyses were not expected to be identical. In the resulting network, nodes represented keywords and links represented their co-occurrence relationships. Larger nodes indicated more frequent keywords, while stronger links represented stronger co-occurrence relationships. Clusters were interpreted as groups of closely related research topics.

#### 2.4.6. Post-Hoc Cardiovascular Analysis

Following the primary bibliometric analysis, a post-hoc exploratory analysis was conducted to assess the representation of cardiovascular health among the included women-only publications. The titles, abstracts, and keywords of the 1345 included publications were searched by two researchers using the Rayyan platform for the terms “cardiovascular,” “CVD,” “heart disease,” “heart failure,” “coronary heart disease,” and “myocardial infarction.” The second researcher then extended the search using the additional terms “cerebrovascular disease” and “stroke.” Publications identified through these searches were reviewed to determine whether cardiovascular health was a primary focus of the research. The number and proportion of cardiovascular-related publications were calculated descriptively.

### 2.5. Sensitivity Analysis

To assess the potential incompleteness of Scopus coverage, particularly for Taiwan-based research published in local journals, the search was replicated in Airiti Library, a major database of Taiwanese academic publications. This analysis was intended to assess whether the Scopus-based retrieval strategy may have missed eligible English-language publications rather than to estimate the overall volume of Chinese-language women’s health research. The Airiti search used the broader terms “Women’s Health” OR “Female Health”. The same inclusion and exclusion criteria were applied to the retrieved records. The results were compared with the Scopus-based findings to assess the potential contribution of eligible English-language publications not captured by Scopus.

### 2.6. Ethical Considerations

This study used bibliographic information obtained from publicly accessible bibliographic databases and did not involve direct interaction with human participants or access to identifiable individual-level health information. Therefore, institutional ethical approval was not required.

## 3. Results

The bibliometric analysis included 1345 publications from 446 journals published between 2015 and 2025. The annual growth rate of publication output was −0.4% over the study period. The average age of the documents was 6.0 years, with an average of 14.52 citations per document. In this study, a total of 3412 authors contributed to the field, and a total of 23 publications were single-authored, contributed by 10 authors. The average number of co-authors per document was 6.64, showing a highly collaborative research environment. International co-authors accounted for 19.48% of all publications. For the bibliographic content, the dataset included 7744 Keywords Plus terms and 3014 author keywords (Table 1).

### 3.1. Publication Trends of Women’s Health Research

**Figure 2:**
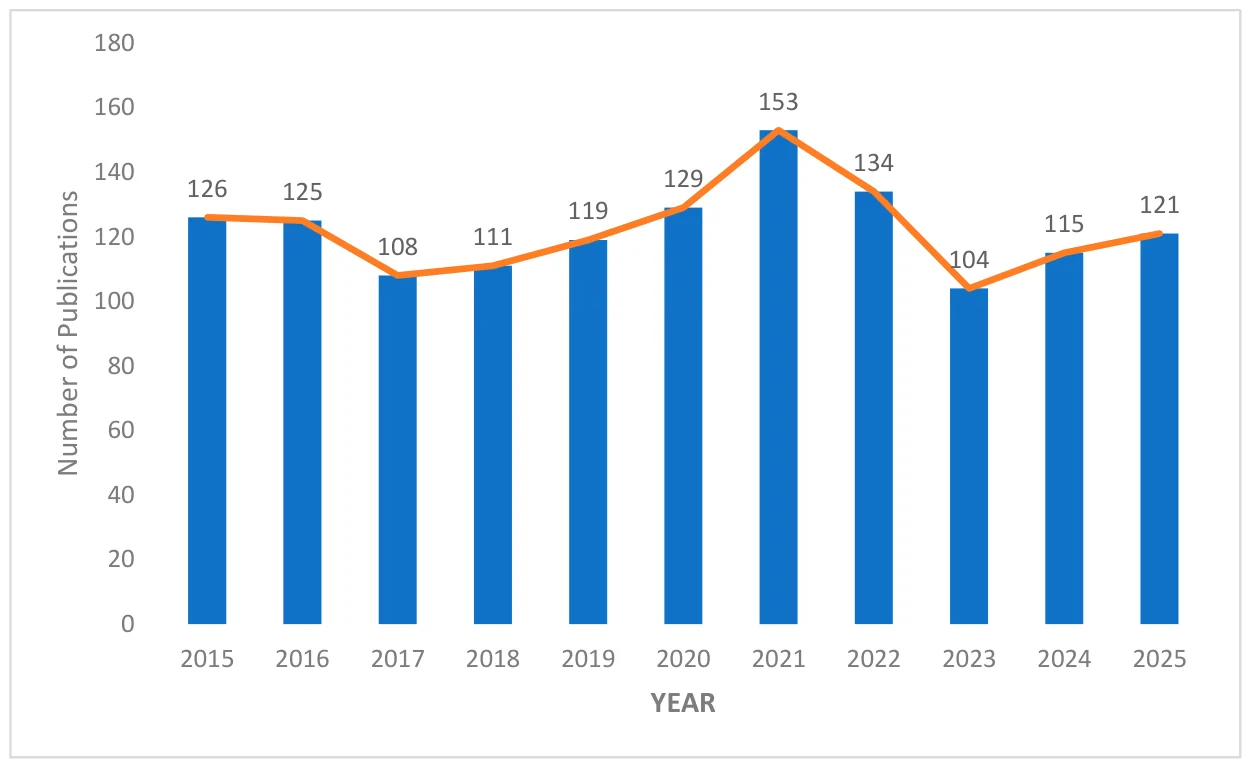
Annual publication trends in women-only health research in Taiwan.

Publication output increased from 126 articles in 2015 to a peak of 153 articles in 2021, followed by a decline in subsequent years (Figure 2). Despite this decline, annual publication output remained above 100 articles throughout the study period, indicating sustained research activity in women-only health research in Taiwan.

### 3.2. International Collaboration

The United States was the leading international collaborator, with 136 collaborative publications. Other major collaborating countries and regions included Australia (35 publications), South Korea (31), the United Kingdom (30), and Hong Kong (23), followed by Japan (20), Canada (16), Singapore (16), Indonesia (11), and India (7) (Table 2). Overall, international co-authorship accounted for 19.48% of the publications.

### 3.3. Institutional Contributions, Secondary Data Sources, and Highly Cited Publications

**Figure 3:**
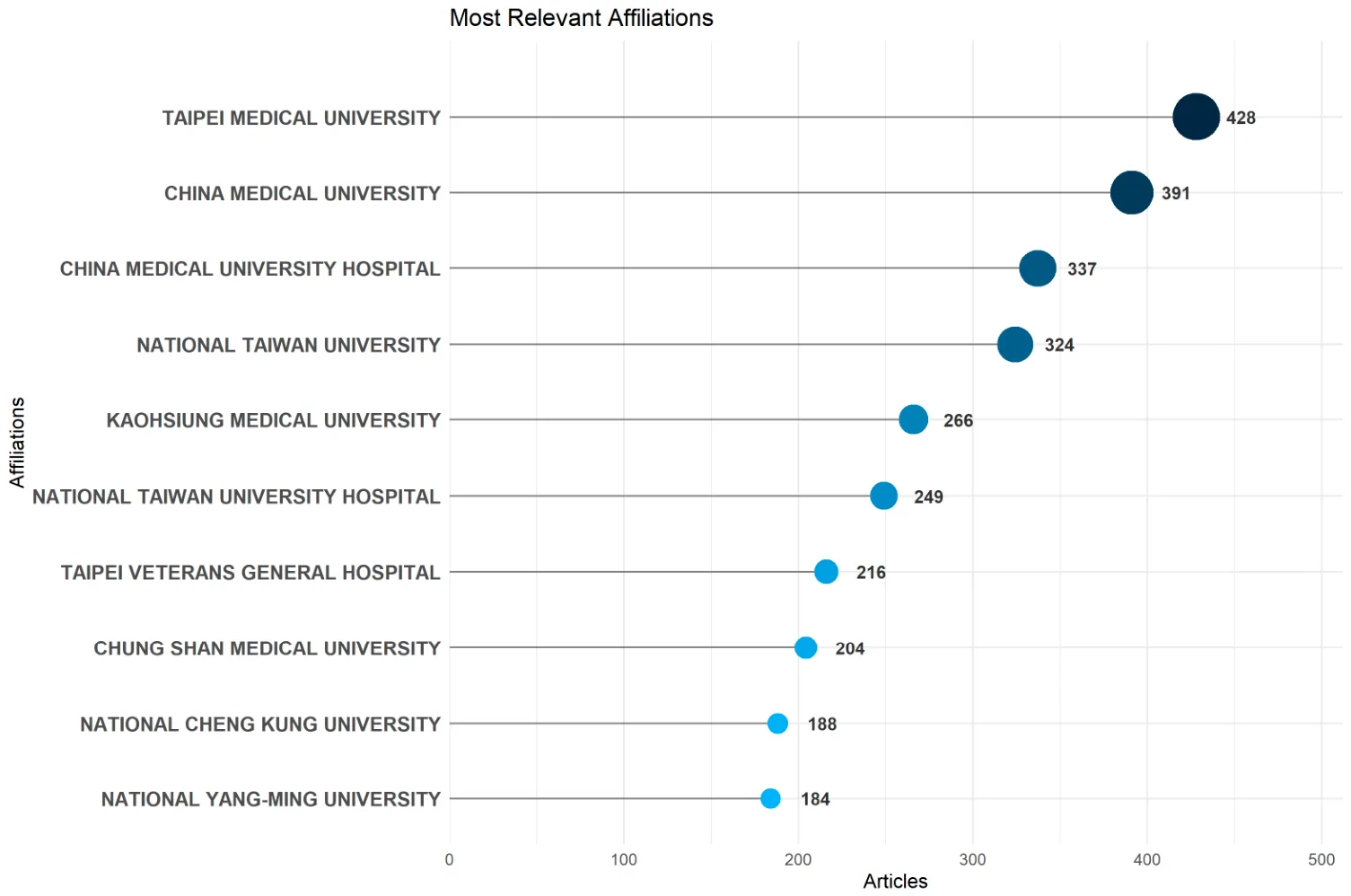
Affiliations with highest number of publications in women-only health.

Several academic and healthcare institutions in Taiwan contributed substantially to women-only health research (Figure 3). The leading institutions included Taipei Medical University (n = 428), China Medical University (n = 391), China Medical University Hospital (n = 337), and National Taiwan University (n = 324), indicating that research capacity was concentrated in these major academic and healthcare institutions. The National Health Insurance Research Database (NHIRD) was the most frequently used secondary dataset (n = 371, 27.6%), followed by the Taiwan Cancer Registry (n = 78, 5.8%) and Taiwan Biobank (n = 33, 2.5%), as shown in Table 3. These datasets were used across a range of research areas, including reproductive and maternal health, cancer, mental health, chronic diseases, genetics, and metabolic health. Other data sources included hospital-based registries and other secondary datasets.

Table 4 presents the most globally cited publications in health research involving only women participants in Taiwan. The most cited article was authored by Yala et al. and published in *Science Translational Medicine* in 2021, with 238 citations and an average of 39.7 citations per year [26]. It was followed by an article by Chen LC et al., published in the *Journal of Affective Disorders* in 2016, with 182 citations and an average of 16.5 citations per year [27]. Yala et al. also published an article in the *Journal of Clinical Oncology* in 2022, which ranked third among the most globally cited articles [28], which garnered 166 total citations and an average of 33.2 citations per year.

### 3.4. Source Publications

**Figure 4:**
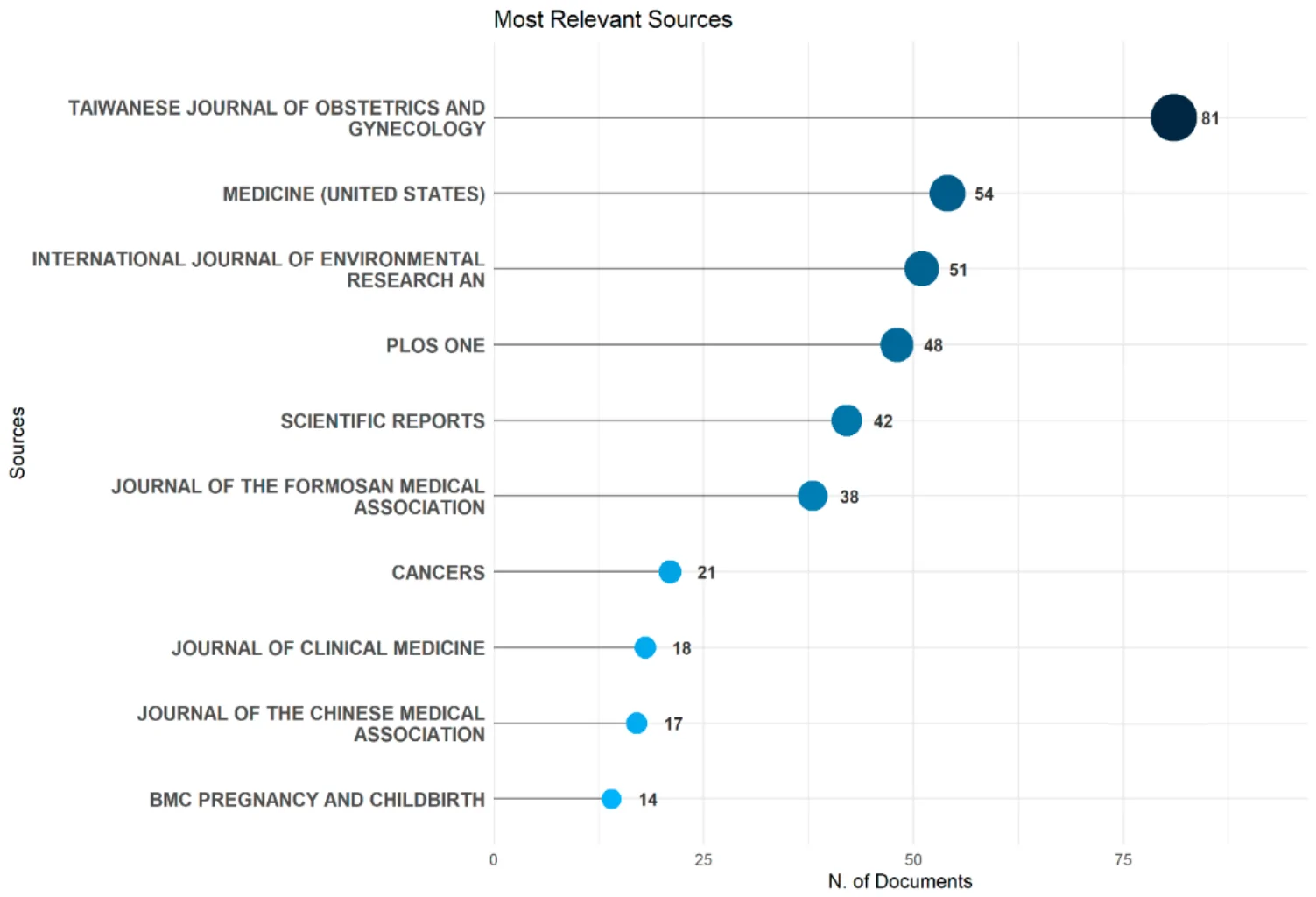
Top 10 journals publishing women’s health research in Taiwan.

The publications were distributed across a broad range of journals, with the 10 most productive journals accounting for a substantial proportion of the literature (Figure 4). The distribution of publication sources spanned multiple areas, including reproductive and maternal health, oncology, chronic diseases, mental health, and public health.

### 3.5. Thematic Map, Keyword Frequency and Research Trend Topics

**Figure 5:**
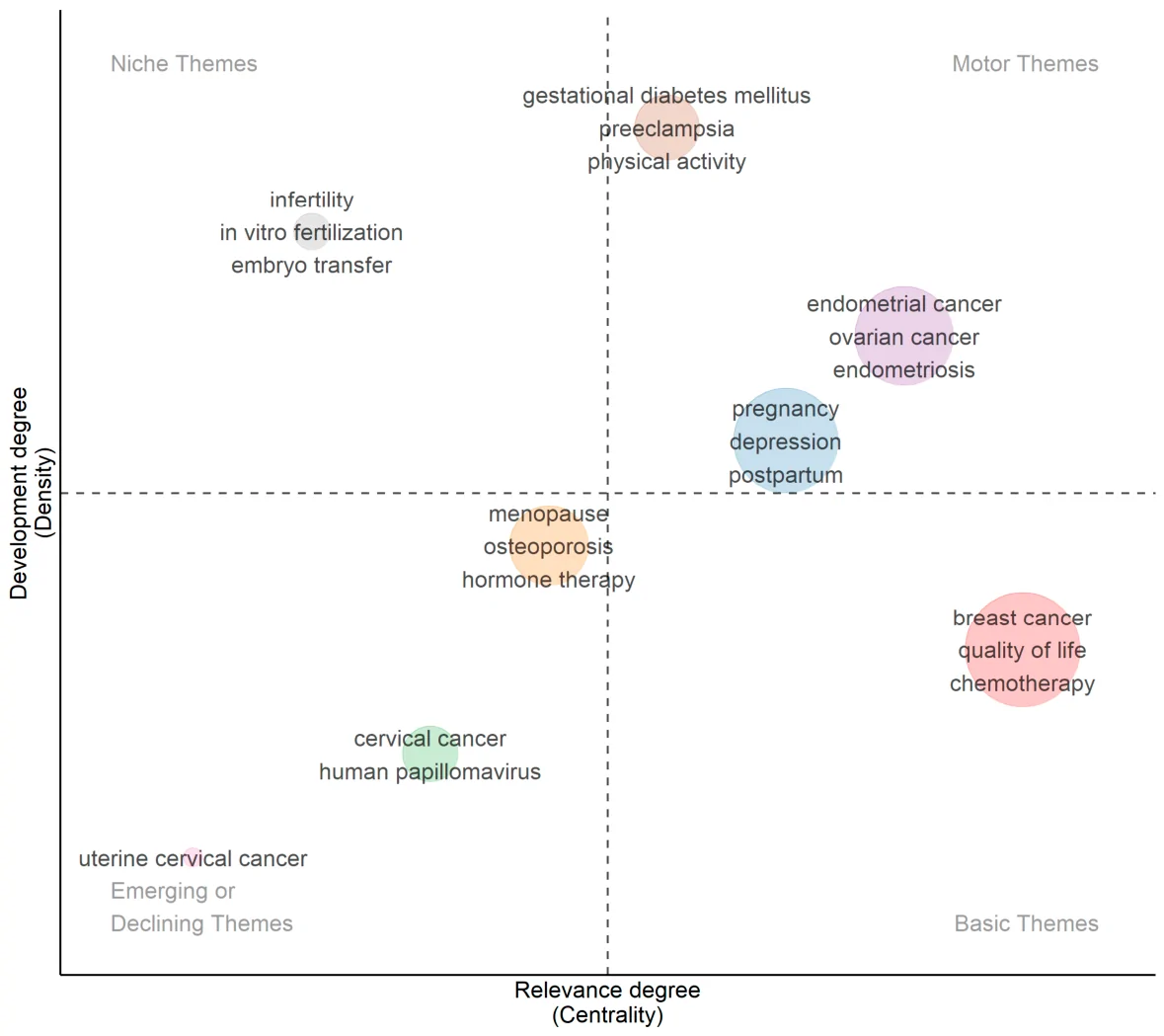
Thematic map from authors’ keywords, excluding generic keywords.

The thematic map based on authors’ keywords identified several major research domains (Figure 5). Motor themes included pregnancy, depression, postpartum health, and gynecological cancers, reflecting themes with relatively high centrality and density. Basic themes included breast cancer, quality of life, and chemotherapy, whereas niche themes included infertility and assisted reproductive technologies. Cervical cancer and human papillomavirus were positioned in the emerging/declining quadrant.

**Figure 6:**
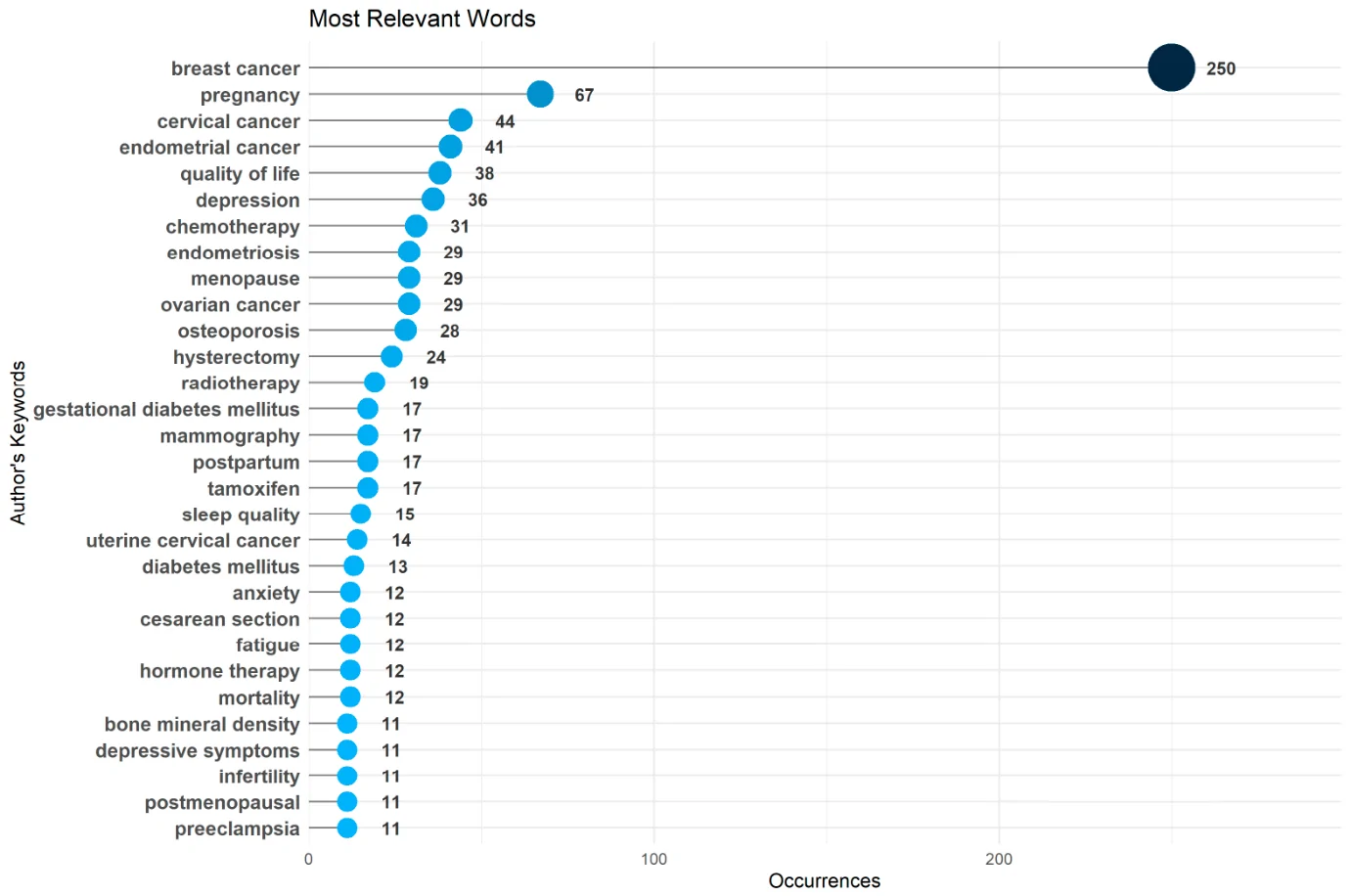
Top 30 most frequently occurring author keywords, excluding generic keywords.

**Figure 7:**
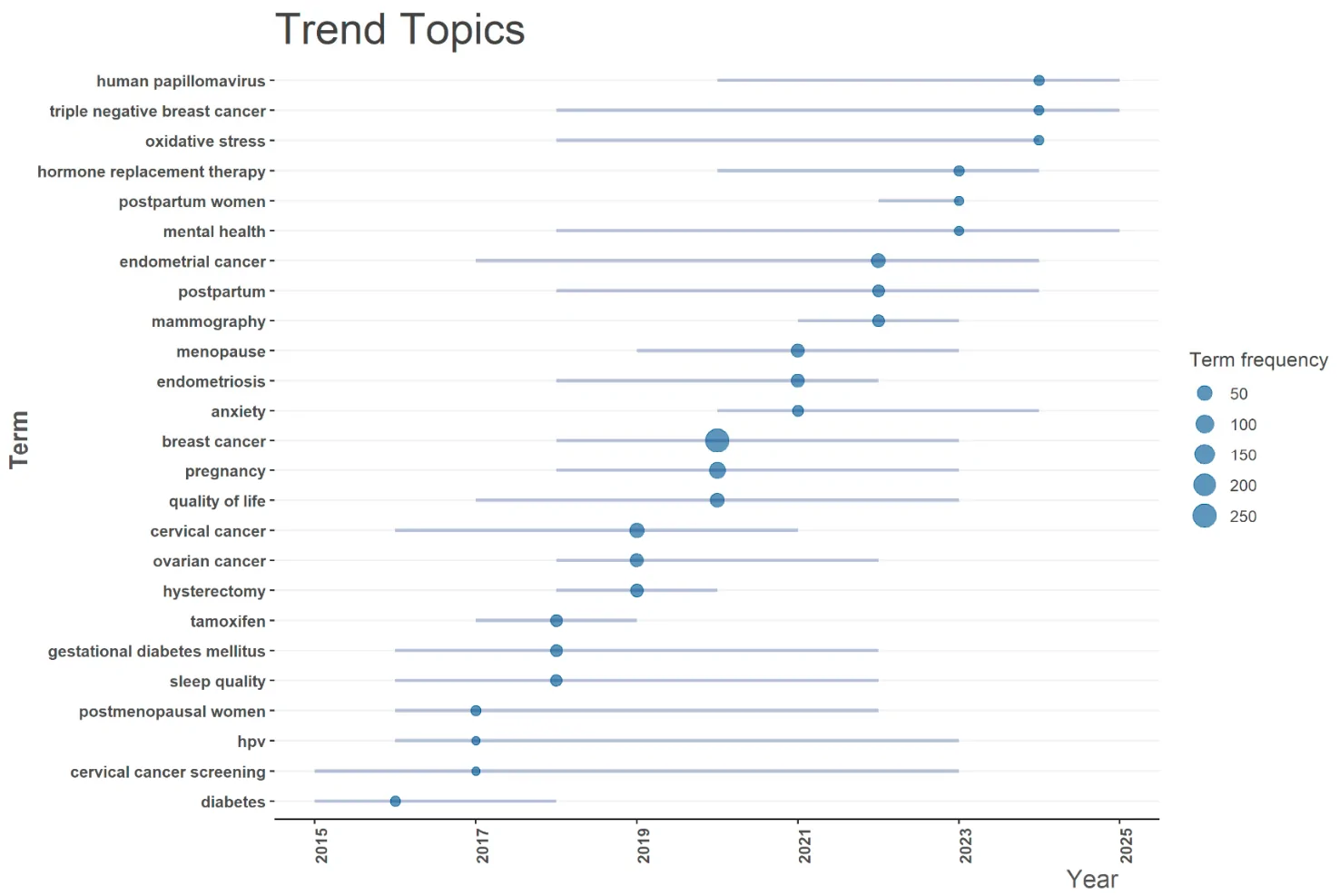
Timeline of research trends based on authors’ keywords, excluding generic keywords.

Analysis of the 30 most frequently occurring author keywords showed that women-only health research in Taiwan primarily focused on reproductive and maternal health, cancer and other chronic diseases, and mental health (Figure 6). Frequently occurring keywords included breast cancer, cervical cancer, pregnancy, depression, endometriosis, and quality of life. The temporal analysis showed changes in research emphasis across the study period (Figure 7). Earlier research prominently featured diabetes, cervical cancer screening, HPV, and postmenopausal women, while breast cancer, pregnancy, quality of life, and gynecological cancers were prominent around 2019–2021. From 2021 onward, topics such as menopause, endometriosis, postpartum health, mental health, and hormone replacement therapy became more prominent. More recent topics, including human papillomavirus, triple-negative breast cancer, and oxidative stress, emerged around 2023–2024. These patterns indicate shifts in research attention across reproductive, oncological, mental health, and postmenopausal health domains over time.

### 3.6. Keyword Co-Occurrence Network

**Figure 8:**
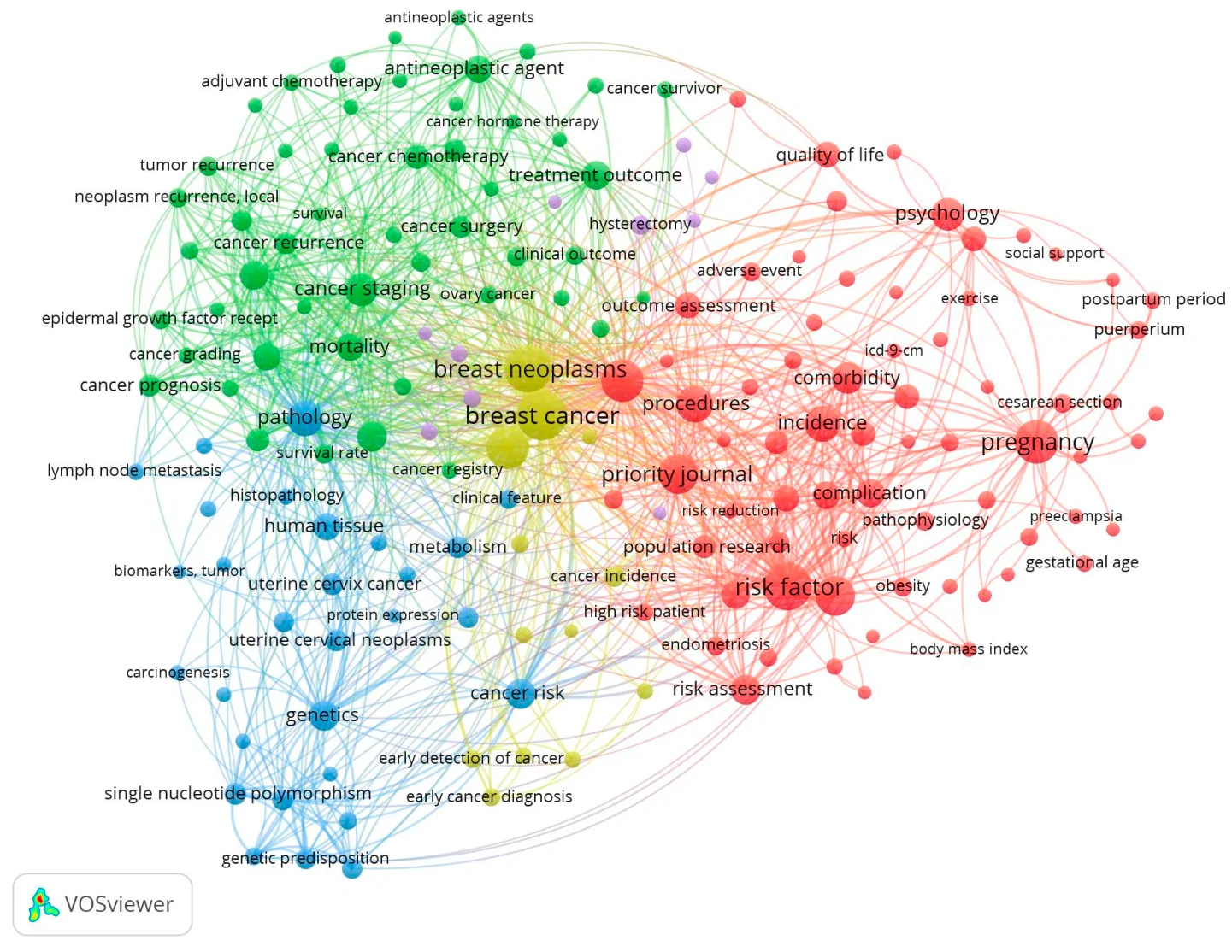
Network visualization of women-only health research in Taiwan (2015–2025), excluding generic keywords. Each keyword is represented by a node, with the color indicating the keyword cluster. Lines connecting the nodes represent the links between keywords, with the distance between two nodes indicating the intensity of their connections.

The VOSviewer analysis initially identified 244 keywords meeting the minimum occurrence threshold of 30. Following manual harmonization and exclusion of generic keywords, 172 keywords were retained. The resulting network comprised five clusters, 10,251 links, and a total link strength of 67,877 (Figure 8). The largest cluster focused on NCDs and related health conditions, including breast cancer and mental health, while a second major cluster centered on maternal and reproductive health. In a post-hoc exploratory analysis of CVD-related publications among women in Taiwan, two researchers screened the included publications using the keywords “cardiovascular,” “CVD,” “heart disease,” “heart failure,” “coronary heart disease,” and “myocardial infarction,” identifying 40 publications. During subsequent review, one researcher identified an additional publication addressing atrial fibrillation, resulting in 41 publications. The same researcher then conducted an additional targeted search using the cardiovascular-related terms “stroke” and “cerebrovascular disease,” which identified a further 9 publications, resulting in a total of 50 CVD-related publications. These 50 publications, representing 3.7% of the 1345 included publications, were classified as primarily focusing on cardiovascular health and were verified by the second researcher to ensure that they met the predefined cardiovascular classification criteria. For uncertain cases, the publication title, abstract, and full text were reviewed as necessary, and any uncertainties were discussed between the researchers and resolved by consensus.

### 3.7. Sensitivity Analysis

A sensitivity analysis was conducted using the local database Airiti Library to assess the potential incompleteness of the Scopus-based retrieval strategy [29]. Using the broad search terms “Women’s Health” OR “Female Health” in Taiwan, 54 records with English-language full texts available were identified. After applying the same eligibility criteria as those used in the primary analysis, 19 articles met the criteria for women-only health research in Taiwan. Of these 19 eligible articles, 4 were also captured in the primary Scopus dataset, whereas 15 were not identified by the primary Scopus search. Thus, the Airiti Library sensitivity analysis identified 15 additional eligible English-language publications beyond those captured by the primary Scopus search. These findings indicate that the Airiti Library search identified additional eligible publications and suggest that reliance on Scopus alone may have resulted in some eligible English-language publications not being retrieved by the primary search strategy. However, given the narrower search scope and limited number of eligible records, these findings do not establish the completeness of Scopus coverage. Chinese-language publications may also remain underrepresented in international bibliometric databases.

## 4. Discussion

To our knowledge, this bibliometric study provides the first Scopus-based bibliometric overview of women-only health research in Taiwan over the 2015–2025 study period. Taiwan’s universal health insurance system and rich population-based databases have supported a strong evidence base for women’s health research, particularly in epidemiological and clinical investigations. By focusing exclusively on studies involving women, this approach captures research priorities and evidence generation that may otherwise be obscured in analyses of mixed-sex populations. Women-only studies can identify sex-specific disease manifestations, treatment responses, reproductive transitions, and social determinants that may be obscured in mixed-sex analyses [30,31].

The thematic analysis revealed that women-only health research in Taiwan has been largely driven by cancer-related, reproductive, and mental health topics. International studies and bibliometric analyses have also shown that research on women’s health is concentrated on reproductive and maternal health, breast and gynecological cancers, and mental health, reflecting long-standing research priorities in these areas and potentially influenced by demographic transitions, population aging, and national screening programs [32,33,34,35]. Globally, breast cancer continues to be a leading cause of cancer-related death among women [36]. The results suggest that Taiwan’s women-only health research agenda is generally consistent with global trends and major health issues affecting women, rather than a unique national pattern. These findings are consistent with the major disease burdens and healthcare priorities affecting women in Taiwan. The prominence of breast and gynecological cancer research likely reflects the substantial public health impact of these conditions, national screening initiatives, and the availability of cancer registry data [12,13]. Similarly, the strong representation of reproductive and maternal health likely reflects Taiwan’s ongoing demographic transition, characterized by persistently low fertility rates and delayed childbearing. As the average maternal age continues to rise [37], concerns regarding fertility, pregnancy outcomes, and women’s reproductive health across the life course have become increasingly important research and policy priorities in Taiwan. The growing visibility of mental health topics further suggests increasing recognition of the psychological dimensions of women’s health, particularly reproductive-related conditions [38], chronic disease management [39], and quality of life [40].

In this study, cardiovascular health represented a relatively small proportion of women-only research in Taiwan, despite cardiovascular disease being a leading cause of death among women globally [7], and in Taiwan [41]. In the post-hoc exploratory analysis, 50 of the 1345 included publications (3.7%) were identified as primarily focusing on cardiovascular health based on keywords including cardiovascular, CVD, heart disease, heart failure, stroke, coronary heart disease, cerebrovascular disease, and myocardial infarction. This finding is consistent with previous reports of limited representation of women in cardiovascular research [5,6]. However, this finding warrants cautious interpretation. Taiwan has conducted several women-only cardiovascular studies [42,43,44], and additional evidence has emerged from sex-stratified analyses of mixed-sex populations [45], which were excluded from our analysis by design. Thus, the 3.7% proportion should be interpreted as a characteristic of the women-only research landscape rather than evidence of inadequate cardiovascular research or an unmet research priority. Bibliometric publication volume reflects research activity and does not necessarily correspond to disease burden, clinical importance, or unmet healthcare needs.

This study involved women-only research outputs from primary and secondary data sources. We included randomized trials and observational studies among these publications. The main sources of primary data were hospital-based and community-based data. Among secondary datasets, the National Health Insurance Research Database was by far the most frequently used, followed by the Taiwan Cancer Registry and the Taiwan Biobank. These findings highlight the importance of Taiwan’s health information infrastructure in supporting women-only health research. The nationwide coverage, large sample sizes, longitudinal follow-up, and linkage capabilities of these databases provide unique opportunities to investigate disease burden, treatment outcomes, healthcare utilization, and population-level determinants of health [9,46]. Numerous studies focusing exclusively on women or analyzing women-only data have utilized these databases [47,48,49]. The substantial reliance on secondary datasets also highlights Taiwan’s capacity to support large-scale epidemiological research that can inform both national and international women’s health research agendas and priorities. Recent nationally representative studies with international visibility have examined the impact of international evidence on menopausal hormone therapy use among Taiwanese women [50]. In addition, the Taiwan Women’s Health Initiative established a women-only cohort between 1993 and 1998, with subsequent follow-up studies generating internationally visible evidence on women’s health [51]. This illustrates how Taiwan’s established research infrastructure and longitudinal cohorts may support the generation of robust evidence on women’s health trajectories.

The analysis of international collaboration patterns revealed that Taiwanese researchers most frequently collaborated with investigators from the United States, Australia, the United Kingdom, South Korea, and Japan. These partnerships likely reflect shared scientific interests, established academic networks, and participation in multinational research initiatives [52]. A previous bibliometric study revealed that international partnerships can have both local and global impacts, enhance research productivity, and improve research reliability [53]. In the present study, international co-authorship accounted for approximately one-fifth of publications, suggesting that opportunities remain to strengthen Taiwan’s integration within global women’s health research networks. Further strengthening international collaboration could provide opportunities to expand research networks and facilitate broader knowledge exchange.

### 4.1. Strengths and Limitations

This study has several strengths. To our knowledge, this is the first bibliometric analysis specifically examining women-only health research in Taiwan and provides a systematic assessment of publication trends, influential contributors, collaboration patterns, dataset utilization, and thematic developments over an eleven-year period. The use of established bibliometric methods and visualization techniques enabled systematic mapping of the research landscape and identification of major thematic patterns. Several limitations should also be acknowledged. First, the use of predefined women’s health keywords may have influenced the relative representation of some research areas. Although the search strategy was intentionally designed to encompass major domains of women’s health across the life course, studies on emerging or less frequently investigated conditions using terminology outside the selected search terms may have been missed. However, a sensitivity analysis using broader generic women’s health terms yielded a similar thematic distribution, suggesting that the predominance of cancer and reproductive health was unlikely to be solely attributable to the search strategy. Second, the analysis was restricted to English-language publications indexed in Scopus and may therefore have excluded relevant studies published in Chinese-language journals or indexed in other databases. Consequently, the present analysis does not capture the full volume of women’s health research conducted in Taiwan. Future bibliometric analyses incorporating both the English- and Chinese-language literature across multiple databases could provide a more comprehensive overview of the research landscape. Third, although indicators such as the h-index and self-citation rate are commonly used in bibliometric studies, they were not included because the present study focused on publication output, citation characteristics, collaboration patterns, data-source utilization, and thematic structure rather than research performance assessment. The findings should therefore be interpreted primarily as a description of the research landscape rather than as a comprehensive assessment of research performance or impact. Another limitation concerns the operational definition of women-only health research. We included only studies conducted exclusively among female participants and excluded mixed-sex studies, including those reporting female-specific or sex-stratified findings. Although this criterion ensured consistency with our study objective, it may have excluded relevant evidence concerning women’s health. Thus, our findings represent research conducted exclusively among women rather than the broader women’s health evidence base in Taiwan. Future studies could separately examine mixed-sex studies with sex-specific findings to provide a more comprehensive assessment. Citation counts may also favor older publications because these articles have had more time to accumulate citations. Therefore, the citation counts and average citations per document reported in this study should be interpreted in the context of publication age. Furthermore, the relatively limited representation of cardiovascular research should be interpreted cautiously because publication volume was not assessed against population-level disease burden or health priorities. Finally, the descriptive nature of this bibliometric study limits causal inference; observed patterns in database use and international collaboration should not be interpreted as evidence that these factors directly influence research productivity, visibility, or health outcomes.

### 4.2. Policy and Research Implications

The findings have several implications for women’s health research and policy planning in Taiwan. First, the concentration of women-only research on cancer and reproductive health indicates sustained attention to established areas of women’s health, whereas cardiovascular health accounted for a comparatively smaller proportion of research output. Although publication volume alone cannot determine whether research activity is proportionate to population health and healthcare needs, this pattern identifies an area that may warrant greater attention in future research priority-setting. Bibliometric evidence could be considered alongside population-level indicators, including incidence, mortality, disability burden, and sex-specific cardiovascular risk, to provide a more comprehensive assessment of research priorities. Such an evidence-informed approach may help broaden research priorities across the life course and support alignment with the broader goals of SDG 3 (Good Health and Well-being) and SDG 5 (Gender Equality). Second, the extensive use of national secondary databases, particularly Taiwan’s population-based health data resources, highlights the importance of existing data infrastructure for women’s health research. Continued development, linkage, and appropriate integration of these resources may facilitate research on women’s health across the life course, including long-term health trajectories, sex-specific risk factors, and interactions among physical, mental, and social health outcomes. Future research could further evaluate how data linkage and integration influence the scope, quality, and policy relevance of women’s health research in Taiwan. Third, the observed level of international collaboration highlights opportunities to further strengthen research networks and facilitate broader knowledge exchange. Such partnerships could support methodological expertise, comparative research, and wider dissemination of findings; however, their effectiveness in strengthening research capacity or informing policy would require assessment beyond bibliometric indicators. Overall, these findings highlight opportunities to broaden the thematic scope of women’s health research, strengthen the use of existing data infrastructure, and expand international engagement. Integrating bibliometric evidence with population health and healthcare needs, research capacity, and policy priorities may help inform more equitable and comprehensive research priorities for women’s health across the life course in Taiwan.

## 5. Conclusions

This study provides a Scopus-based bibliometric overview of women-only health research in Taiwan from 2015 to 2025. Publication output increased from 126 articles in 2015 to a peak of 153 in 2021, followed by a decline, while remaining above 100 articles annually throughout the study period, indicating sustained research activity. Cancer, reproductive health, and mental health were major areas of research activity, whereas cardiovascular health accounted for a comparatively smaller proportion of the literature. The extensive use of national health databases and international collaboration further characterized Taiwan’s women’s health research landscape. These findings provide an evidence-informed basis for future research priority-setting and planning, particularly efforts to broaden research across women’s health conditions and life-course stages while considering research activity alongside population health and healthcare needs. Integrating bibliometric evidence with population health priorities may support a more balanced and comprehensive women’s health research agenda in Taiwan, while informing broader efforts aligned with SDG 3 (Good Health and Well-being) and SDG 5 (Gender Equality).

## Figures and Tables

**Table 1:** Main information about final bibliometric data.

Description	Result
Timespan	2015:2025
Sources (Journals)	446
Documents	1345
Annual Growth Rate %	−0.4
Document Average Age	6.0
Average citations per doc	14.52
Keywords Plus (ID)	7744
Author’s Keywords (DE)	3014
Authors	3412
Authors of single-authored docs	10
Single-authored docs	23
Co-Authors per Doc	6.64
International co-authorships %	19.48

**Table 2:** International collaboration by country.

Country	Number of Articles
United States of America	136
Australia	35
South Korea	31
United Kingdom	30
Hong Kong	23
Japan	20
Canada	16
Singapore	16
Indonesia	11
India	7

**Table 3:** Most frequently used secondary datasets in women-only health research in Taiwan (2015–2025) and their common research areas.

Rank	Secondary Dataset	N (%)	Common Research Areas
1	National Health Insurance Research Database, including the Longitudinal Health Insurance Database (LHID)	371 (27.6)	Reproductive and maternal health, cancer, mental health, chronic diseases
2	Taiwan Cancer Registry	78 (5.8)	Breast cancer, cervical cancer, gynecologic cancers
3	Taiwan Biobank	33 (2.5)	Genetics, lifestyle factors, metabolic health, menopause

**Table 4:** Most globally cited publications.

Paper	Doi	Total Citation	Average Total Citation per Year
YALA A, 2021, SCI TRANSL MED	10.1126/scitranslmed.aba4373	238	39.7
CHEN LC, 2016, J AFFECTIVE DISORD	10.1016/j.jad.2015.10.030	182	16.5
YALA A, 2022, J CLIN ONCOL	10.1200/JCO.21.01337	166	33.2
SUNG H, 2015, J NATL CANCER INST	10.1093/jnci/djv107	141	11.8
LI SW, 2017, FRONT MICROBIOL	10.3389/fmicb.2017.00965	136	13.6
KOK VC, 2015, INT J GYNECOL CANCER	10.1097/IGC.0000000000000454	111	9.20
HUNG TH, 2016, TAIWANESE J OBSTET GYNECOL	10.1016/j.tjog.2016.06.016	104	9.50
CHANG SM, 2016, J ADV NURS	10.1111/jan.12836	98	8.90
TSAI M, 2020, ENVIRON INT	10.1016/j.envint.2020.105850	97	13.9
HSIEH CJ, 2019, SCI TOTAL ENVIRON	10.1016/j.scitotenv.2018.08.149	96	12.0

## Data Availability

The complete Boolean search strategy is reported in the Methods section. Supplementary File provides the exclusion of generic words used in the bibliometric analyses. The bibliographic records were retrieved from Scopus and are subject to the database licensing conditions. Additional documentation is available from the corresponding author upon request.

## Supplementary Materials

The following supporting information can be downloaded at: https://www.mdpi.com/article/10.3390/healthcare14172842/s1, File S1: Lists of generic author keywords excluded from Figure 5, Figure 6, Figure 7 and Figure 8.
