# Supplementary material for: Mapping Women-Only Health Research in Taiwan: A Bibliometric Analysis of Research Trends, Collaboration, and Thematic Clusters (2015–2025)
Source: Healthcare (Basel). 2026 Sep 3;14(17):2842. doi: 10.3390/healthcare14172842 (PMC13566305; doi:10.3390/healthcare14172842)
Supplement: Supplementary file 1 [file healthcare-14-02842-s001.zip › healthcare-4490684-supplementary.pdf]

Supplementary Information

File S1. Lists of generic author keywords excluded from Figures 5–8.

A: Lists of generic authors' keywords that were excluded in Figure 5.

Epidemiology, women, database, national health insurance research database, Taiwan, female, age, overall survival, incidence, risk, DNA methylation biomarker, polymorphism, genotype, pregnant women, traditional Chinese medicine, genetic variants, single nucleotide polymorphism, cohort study, recurrence, self-efficacy, screening, Chinese herbal medicine.

B: Lists of generic authors' keywords that were excluded in Figure 6.

Taiwan, women, cohort study, epidemiology, polymorphism, traditional Chinese medicine, pregnant women, database national health insurance research, database, survival, genotype, prognosis, social support, female, laparoscopy, risk factors.

C: Lists of generic authors' keywords that were excluded in Figure 7.

Pregnant women, screening, national health insurance research database, Taiwan, women, cohort study, epidemiology, polymorphism, nurses, laparoscopy, doing the month, social support, and risk factors.

D: Lists of generic authors' keywords that were excluded in Figure 8.

Adult, age*, article, Asian* case-control stud*, Chinese medicine, clinical article, clinical trial, cohort analysis, cohort stud*, comparative study, confidence interval, controlled study, cross-sectional studi*, data base, demography, epidemiology, ethnology, female*, groups by age, hazard ratio, health insurance, health survey, human*, kaplan meier*, logistic models, longitudinal studi*, major clinical study, medical record review, middle aged, multicenter study, multivariate analysis, national health insurance, observational study, odds ratio, prediction, predictive value, pregnant women, propensity score, proportional hazard ratio*, prospective studi*, qualitative research, questionnaire, registri*, receiver operating characteristic, retrospective studi*, statistical model, statistic*, surveys and questionnaires, Taiwan*, very elderly, women*, young adult.
